# Development of the Finnish neurological function testing battery for dogs and its intra- and inter-rater reliability

**DOI:** 10.1186/s13028-018-0408-2

**Published:** 2018-09-17

**Authors:** Anna Fredrika Boström, Heli Katariina Hyytiäinen, Petteri Koho, Sigitas Cizinauskas, Anna Katrina Hielm-Björkman

**Affiliations:** 1 Department of Equine and Small Animal Medicine, Faculty of Veterinary Medicine, University of Helsinki, P.O. Box 57, 00014 Helsinki, Finland; 2 Rehabilitation Orton, Tenholantie 10, 00280 Helsinki, Finland; 3 Small Animal Hospital Aisti, Virtatie 9, 01600 Vantaa, Finland

**Keywords:** Inter-rater reliability, Intra-rater reliability, Motor function, Neurology, Outcome measure, Physiotherapy, Testing battery, Validity

## Abstract

**Background:**

The Finnish neurological function testing battery for dogs (FINFUN) was developed to meet the increasing demand for objective outcome measures in veterinary physiotherapy. The testing battery should provide consistent, reproducible results and have established face and content validity. Internal consistency and intra- and inter-rater reliability of the FINFUN were also investigated.

**Results:**

The FINFUN comprised 11 tasks: lying, standing up from lying, sitting, standing up from sitting, standing, proprioceptive positioning, starting to walk, walking, trotting, walking turns and walking stairs. A score from 0 to 4, (0: unable to perform task, 4: performing task with normal motor function) was given for each task, the maximum score being 44. Twenty-six dogs were filmed when performing the FINFUN. Seven observers scored the performances from the video recordings. The FINFUN was considered to have appropriate face and content validity based on a pilot study, clinical experience and critical reflection of the development process. Its internal consistency was excellent, with no Cronbach’s alpha values below 0.922. The intra-rater reliability for total score of experienced observers was almost perfect: 0.999 (observer 1) and 0.994 (observer 2). The inter-rater reliability for both experienced and novice observers’ total scores was also almost perfect (0.919–0.993). Analysis of each individual task showed substantial intra-rater and inter-rater agreement for the tasks “lying” and “sitting”.

**Conclusions:**

The FINFUN is an objective, valid and reliable tool with standardized scoring criteria for evaluation of motor function in dogs recovering from spinal cord injury.

**Electronic supplementary material:**

The online version of this article (10.1186/s13028-018-0408-2) contains supplementary material, which is available to authorized users.

## Background

Dogs recovering from different grades of paralysis caused by disease or trauma to the nervous system are seen daily in veterinary hospitals and rehabilitation practices [1, 2]. Veterinary physiotherapy is an important part of the modern treatment regime for these neurological patients [3]. Maintenance and enhancement of functional ability in affected dogs is important to ensure rapid recovery and a good quality of life, and physiotherapy has been shown to be beneficial for dogs recovering from intervertebral disc disease [3–5], fibrocartilaginous embolism [6] and degenerative myelopathy [7].

In human neurology, the physiotherapist evaluates and trains everyday motor function with the aim of the individual being able to continue with work and activities of daily living [8]. Objective evaluation of motor function and change over time is important and requires accurate and repeatable measurements and reliable instruments [8]. Human neurological physiotherapy has several established, validated and reliable functional outcome measures for patients with spinal cord injury and stroke that are used in both clinical practice and research [8–12].

The demand for efficient, safe and evidence-based physiotherapy strategies is increasing also in veterinary medicine, creating a need for sensitive validity and reliability testing instruments to assess the recovery from and effects of different interventions [13]. To the authors’ knowledge, there are no functional testing batteries that evaluate overall motor function of dogs with neurological disease. Overall motor function comprises functional everyday tasks like sitting and standing, transitions from lying or sitting to standing, from standing to walking and ambulation at different speeds. No testing battery so far include voluntary motor functions progressing towards more advanced locomotion and activities of daily living, and is as well convenient to use in both clinical practice and research. Objective outcome measures should be validated and evaluated for internal consistency and intra-and inter-rater reliability [12, 14]. Face validity considers whether users or experts agree that the instrument is measuring what it is intended to measure, and content validity is the degree to which all tasks in the measure assess the same domain of interest [12]. Internal consistency means that all tasks in the instrument measure the same attribute [12, 14]. Intra-rater reliability is the degree to which scores on the instrument obtained by one trained observer agree with the scores obtained when the same observer administers the measure on another occasion [12]. Inter-rater reliability is the degree to which scores on the instrument obtained by one trained observer agree with the scores obtained by another trained observer [12, 14].

Validated outcome measures are consistently used in the experimental setting to quantify the progress in the animal’s locomotor function [15, 16], but implementing them in clinical practice is not straightforward [17]. In small animal clinical practice, the veterinary modifications of the human Frankel score for spinal cord injury are frequently used for injury classification and outcome determination, but it does not quantify walking [18–21]. The Texas Spinal Cord Injury Score (TSCIS) was created to provide reliable measurements for location and degree of injury as well as to determine outcome in dogs with spinal cord injury [21], but it is limited by the functional evaluation of the ability to walk [21]. Previous studies present useful validated methods to quantify both the ability to walk and the quality of walking [17, 22–24], but these scales do not include any other components of the dog’s functional ability.

The aim of this study was to develop a neurological function testing battery that would measure overall motor function in canine patients. The testing battery would provide consistent, reproducible results independent of neurological disease (i.e. intervertebral disc disease or fibrocartilaginous embolism). The testing battery would need to be cost-effective and convenient to use by veterinary physiotherapists in both clinical and research settings. Further aims were to establish the face and content validity of the testing battery and to investigate its internal consistency and intra- and inter-rater reliability.

We hypothesized that the Finnish neurological function testing battery for dogs (FINFUN) would be valid for assessment of motor function in dogs with neurological disease and that it would display high internal consistency and intra- and inter-rater reliability.

## Methods

### Development of the test

The FINFUN was designed based on a human functional outcome measure, the Motor Assessment Scale [11], the Basso, Beattie, Bresnahan (BBB) Scale for spinal cord injured rats [15], the five recovery stages in dogs with acute spinal cord injuries [22] and the clinical experience of the research team. The general instructions and scoring criteria of the FINFUN can be found in Additional file 1. The FINFUN consists of 11 tasks of progressive difficulty; ‘lying’, ‘standing up from lying’, ‘sitting’, ‘standing up from sitting’, ‘standing’, ‘proprioceptive positioning in affected limbs’, ‘start to walk from standing’, ‘walking’, ‘running’, ‘walking turns’ and ‘walking stairs’.

In each task, the dog´s performance is given a numeric score from 0 to 4, where 0 indicates that the dog is unable to perform the task at all and 4 indicates that the dog is able to perform the complete task with normal motor function or with motor function at the level prior to disease/injury. Two animal physiotherapists (AB and HH) and an ECVN Diplomate (SC) developed the scoring criteria. To ensure consistency in the scoring process, each task is described thoroughly and the criteria for each score in the tasks are specified (Additional file 1).

The testing battery includes a section for comments, where information relevant to the assessment (method of support, method of motivation, medication) can be recorded. This section makes it possible to distinguish between the dog not being able to perform a task physically or just being restricted by support, motivation or the surgeon’s restrictions. To ensure standardized scoring, the FINFUN criteria are additionally accompanied by general instructions for use, specifying equipment and environment requirements as well as providing instructions regarding assistance and motivation of the tested dogs. The scoring time is approximately 15 min.

The FINFUN was validated in both the English and Finnish languages. The general instructions and scoring criteria were created in English and translated to Finnish by a native speaker for the Finnish-speaking observers. The text was then translated back to English and checked by a native speaker from the English language editing services.

A pilot study on intra- and inter-rater reliability was undertaken on 10 dogs with different grades of paralysis. The results showed the FINFUN to have excellent intra- and inter-rater reliability [25]. After the pilot study, one author (AB) used the FINFUN in clinical practice and the scoring criteria were further adjusted according to clinical experience and critical reflections with the observers in the pilot study.

### Observer training

Seven observers volunteered for this study. They were all trained human physiotherapists, specialized in animal physiotherapy. They had all been involved in the pilot study and were thus familiar with the testing battery. Two of the observers were considered experienced, i.e. had worked with neurological patients daily during the last 2 years, and the remaining five were considered novices, i.e. had worked with neurological cases only occasionally. The observers received training in the use of the testing battery, and they practiced the evaluation process both live and from video recordings. The observers were instructed to familiarize themselves with the test criteria thoroughly beforehand. The observers scored the performances from the video recordings of at least eight dogs on their own to establish a routine in the scoring process. The scoring process was revised on the test date, and the observers had the opportunity to ask questions about the testing battery itself or the scoring process before the actual study started. The performances used in the training were not included in the study.

### Study protocol

Twenty-six dogs of different breeds recovering from spinal cord injury caused by intervertebral disc disease, fibrocartilaginous embolism, arachnoidal cyst or neoplasia, were referred to physiotherapy (AB) by the treating veterinary surgeon, where they were evaluated using the FINFUN. The evaluation was part of their agreed routine physiotherapy assessment carried out by an animal physiotherapist (AB). Ethical approval was not required as the evaluation was carried out during standard clinical practice. The dogs were filmed (with owner consent) from the front, behind and both sides while performing the different tasks using a digital video camera. The video recordings were stored on a computer. All observers, except AB, were blinded to the diagnosis and background data of the dogs, as the included dogs had been her patients. All observers consented to patient confidentiality by signing the FINFUN scoring sheet once the scoring was completed. The video recordings were shown to the observers twice and the seven observers evaluated the dogs’ performances from the video recordings according to the FINFUN scoring criteria.

### Inter- and intra-rater reliability

The five novice and two experienced observers scored the dogs from the video recordings according to the test criteria on the same occasion, blinded to each others´ scores. Inter-rater reliability was evaluated for each individual task as well as for the FINFUN sum score for novice and experienced observers separately.

The two experienced observers scored the dogs from the video recordings according to the test criteria on two different occasions with a 3-week interval. The observers were blinded to each others’ and the previous scores. Intra-rater reliability was evaluated for each individual task as well as for the FINFUN sum score.

### Statistical analysis

Mean and standard deviation were used to summarize the descriptive data of the studied dogs. The internal consistency was tested using Cronbach’s alpha. The intra- and inter-rater reliability was analyzed using Intra-class correlation coefficient (ICC), two-way mixed model and absolute agreement, with a confidence interval of 95%. The reliability was reported as follows: slight agreement: 0.01–0.20, fair agreement: 0.21–0.40, moderate agreement: 0.41–0.60, substantial agreement: 0.61–0.80, almost perfect agreement: 0.81–1.00 [26]. SPSS, version 19 (IBM, New York, NY, USA) was used in the analysis.

## Results

### Animals

Of the 26 evaluated dogs, 10 were female and 16 male. Their mean age was 5.0 ± 2.2 years and their mean weight 13.5 ± 9.9 kg. The breed, sex, age, weight, diagnosis and mean, standard deviation (SD) and range of scores for each dog are displayed in Table 1. Magnetic resonance imaging or myelography confirmed the diagnosis in all dogs, and 22 dogs had surgical hemilaminectomy and 2 dogs dorsal laminectomy prior to the physiotherapy referral.

**Table 1:** Descriptive information on the studied dogs

Dog	Breed	Sex	Age (years)	Weight (kg)	Diagnosis/treatment	Mean score (SD)	Range score
1	Mixed	F	8	25.1	T12-L1 disc extrusion, hemilaminectomy	20.3 (2.0)	18–23
2	Dachshund, short-haired	M	5	12.0	T12-13 disc extrusion, hemilaminectomy	9.7 (1.6)	7–12
3	Mixed	M	1	8.0	L1-2 disc extrusion, hemilaminectomy	8.6 (1.7)	5–10
4	Welsh corgi cardigan	F	6	16.5	T12-13 disc extrusion, hemilaminectomy	24.6 (1.6)	22–26
5	Bernese mountain dog	M	7	49.3	L7-S1 disc protrusion, dorsal laminectomy	36.4 (1.7)	34–38
6	Pinscher	F	4	15.0	T11-13 disc extrusion, hemilaminectomy	34.0 (1.6)	32–36
7	Miniature poodle	M	3	7.5	L4-5 disc extrusion, hemilaminectomy	24.1 (2.0)	21–27*
8	Coton de tulear	F	4	5.0	L1-2 disc extrusion, hemilaminectomy	28.9 (1.2)	27–31
9	Dachshund, short-haired	M	9	11.0	T13-L1 disc extrusion, hemilaminectomy	33.6 (1.5)	32–36
10	Tibetan terrier	M	7	12.6	T12-13 disc extrusion, hemilaminectomy	35.6 (2.4)	33–40*
11	Cocker spaniel	F	6	15.8	T12-13 disc extrusion, hemilaminectomy	15.9 (2.1)	14–19
12	French bulldog	M	4	13.8	L1-2 disc extrusion, hemilaminectomy	23.1 (0.9)	22–24
13	Dachshund, wire-haired	M	3	10	T13-L1 disc extrusion, hemilaminectomy	28.3 (1.7)	27–31
14	Dachshund, wire-haired	F	4	5.6	T12-13 disc extrusion, hemilaminectomy	31.9 (0.9)	31–33
15	Mix	F	9	6.2	T12-13 disc extrusion, hemilaminectomy	27.1 (1.6)	24–29
16	Dachshund long haired	M	8	7.5	T12-13 disc extrusion, hemilaminectomy	13.7 (1.0)	12–15
17	Coton de tulear	M	7	8.0	L1-2 disc extrusion, hemilaminectomy	31.0 (1.6)	29–34
18	Dachshund, wire-haired	M	4	5.9	T12-13 disc extrusion, hemilaminectomy	30.9 (2.5)	27–35*
19	Dachshund, short-haired	F	4	8.0	L4-5 disc extrusion, hemilaminectomy	28.7 (0.8)	28–30
20	French bulldog	M	5	16.5	T5-7 arachnoidal cyst, dorsal hemilaminectomy	31.7 (1.6)	30–34
21	Saluki	M	6	28.0	Fibrocartilaginous embolism, conservative treatment	6.4 (1.4)	4–8
22	Dachshund, wire-haired	M	3	12.0	T12-13 disc extrusion, hemilaminectomy	29.4 (1.0)	28–31
23	Doberman	F	5	29.3	T12-L1 osteochondromatosis, hemilaminectomy	25.3 (1.8)	23–27
24	Mixed	F	2	4.4	L1-2 disc extrusion, hemilaminectomy	10.3 (2.6)	7–14*
25	Dachshund, wire-haired	M	3	9.6	T12-13 disc extrusion, hemilaminectomy	37.0 (2.3)	33–40*
26	Löwchen	M	2	7.5	T12-13 disc extrusion, conservative treatment	15.1 (0.9)	14–16

The breed, age, weight and mean ± standard deviation (SD) and range for the given scores for each dog with all observers’ scores considered. Dogs with a wide range in the scores (> 5 points) are indicated with an asterisk (*)

*F* female, *M* male

### Face and content validity

The FINFUN was considered to meet the criteria for good face and content validity. The novice observers agreed with the test developers that the testing battery covered the most essential components for evaluation of functional ability in dogs with neurological disease hence the criterion for good face validity was met. The criteria for the content validity was met, as there was consensus amongst all participants in this study, that each item measured motor function relevant for dogs with neurological disease. This was confirmed by clinical observations showing that dogs with higher functional ability (walking or running for longer distances or requiring less support) got higher scores.

### Internal consistency

All of the observers showed excellent internal consistency, with none below 0.922. Excluding one task at a time from the analysis showed that exclusion of ‘lying*’* (task 1), ‘sitting’ (task 3) and ‘proprioceptive positioning’ (task 6) would increase Cronbach’s alpha for all observers, albeit not enough to alter the internal consistency significantly.

### Inter-rater reliability

The results for the inter-rater reliability are shown in detail in Table 3. The inter-rater reliability between the experienced observers’ total score was almost perfect (ICC 0.993, 95% CI (0.984–0.997)). The agreement ranged from substantial to almost perfect for the separate tasks (0.705–0.993), with ‘lying’ and ‘sitting’ being the tasks with low ICC values and large standard error measures (Table 3). Inter-rater reliability between novice observers’ total scores was almost perfect (ICC (3.5) 95% CI 0.993 (0.988–0.997)) and substantial for ‘sitting’ (95% CI 0.697 (0.465–0.848)). Inter-rater reliability for all observers’ total scores was almost perfect (ICC (3.7) 95% CI 0.996 (0.993–0.998)) and was almost perfect for all tasks, except ‘*sitting*’, for which it was substantial (0.793).

### Intra-rater reliability

Intra-rater reliability for observer 1 total score was almost perfect ICC 0.996, 95% CI (0.991–0.998), ranging from substantial to almost perfect for the separate tasks 0.668–0.957. The observer 2 intra-rater reliability was almost perfect ICC (3.2) 0.994, 95% CI (0.981–0.998) for the total score and for all separate tasks, except ‘sitting’, for which it was moderate 0.464 (− 089 to 0.748) (Table 2).

**Table 2:** Intra-rater reliability for individual tasks and sum score of the testing battery

Task	Intra-rater correlation observer 1 (95% CI)	Intra-rater correlation observer 2 (95% CI)
Lying	0.943 (0.873–0.974)	1.000
Standing up from lying	0.957 (0.904–0.981)	0.979 (0.952–0.991)
Sitting	0.668 (0.224–0.855)	0.464 (− 089 to 0.748)
Standing up from sitting	0.938 (0.861–0.972)	0.917 (0.770–0.966)
Standing	0.976 (0.946–0.989)	0.933 (0.844–0.971)
Proprioceptive positioning	0.988 (0.974–0.995)	0.961 (0.915–0.983)
Start to walk from standing	0.986 (0.970–0.994	0.986 (0.970–0.994)
Walking	0.989 (0.975–0.995)	0.990 (0.977–0.995)
Running	0.984 (0.966–0.993)	0.994 (0.987–0.997)
Walking turns	0.983 (0.961–0.993)	0.978 (0.952–0.990)
Walking stairs	0.976 (0.948–0.989)	0.984 (0.964–0.993)
Total score	0.996 (0.991–0.998)	0.994 (0.981–0.998)

The intra-class correlation coefficient and confidence intervals for the two experienced observers (observer 1 and observer 2). Data are shown for each individual task and for the FINFUN total score

## Discussion

The FINFUN was designed to meet the demand for an objective, validated and reliable functional outcome measure in veterinary physiotherapy. This study shows the FINFUN to be valid and reliable between observers and to provide reproducible results when dogs with different grades of paralysis are assessed from video recordings.

### Development of the test

The development of functional testing batteries must be transparent and well described [13]. This report includes detailed information regarding the development of the testing battery and the educational level, experience and training of the observers. Both human and veterinary outcome measures were used in the development process of the FINFUN [11, 15, 22]. The MAS evaluates task-related interventions in human patients with acute stroke using simple scoring criteria and the tasks are separate actions, which enable them to be used as separate entities based on the information required [11]. These features were desirable in the FINFUN. The ordinal scale used in the FINFUN was chosen according to Olby et al. [22], and the test criteria regarding movement quality were determined based on the in the BBB Locomotor Rating Scale [15].

The FINFUN consists of activities of daily living that are applicable to any pet dog and that progress from easier tasks to more challenging ones. ‘Start to walk from standing’ (task 7) was included as a separate task from walking, as dogs with upper motor neuron lesion may succeed in standing due to normal or increased extensor tone in the hind limbs [27], but they cannot initiate movement or take weight-bearing steps. During activities of daily living the dogs need to move safely and independently through turns, hence walking turns were included. It was challenging to motivate dogs to walk a figure of eight, resulting in a risk for the handler interfering with a dog’s performance. This task showed, however, good reliability and was easily assessed by the observers.

‘Running’ (task 9) and ‘walking stairs’ (task 11) require strength, causing some physical stress to the patient. Dogs recovering from trauma or spinal surgery may not be permitted to run or walk stairs for several weeks postoperatively. Including such tasks in the FINFUN could therefore be questioned, although many households require running and walking stairs for independent locomotion [28]. The FINFUN scoring criteria takes this into account and allows a dog to receive some points for stair climbing and running if it is able to perform the tasks with strong support. Additionally, the assessor may use the comments section to record whether the dog is not yet permitted to perform these tasks due to postsurgical restrictions. The dogs in this study were considered fit enough by their veterinary surgeon to perform all of the tasks. The owners were informed that these activities are not permitted in the home environment at this point of recovery, and they were allowed to withdraw their dog from the running or stair-climbing tasks.

### Face and content validity

The thorough development process, including the translation, the pilot study and the clinical experience with critical reflection of the FINFUN in relation to already reported functional tests, contributed to sufficient face and content validity. The FINFUN users and the small animal neurologist in this study considered the FINFUN to measure overall motor function. Further investigation is needed to determine whether the FINFUN provides results consistent with those of another validated measure (criterion validity) [12, 14].

### Internal consistency

The results show appropriate internal consistency, indicating that the FINFUN measures what it is intended to measure. A high Cronbach’s alpha is considered to increase the reliability of a measure [29]. On the other hand, a high alpha is not always desired because closely correlated tasks may suggest redundancy, and tasks very similar to each other could be considered for exclusion from the testing battery [30]. However, all of the original tasks were maintained in the FINFUN because an extensive measure is more reliable than a more compact one as it increases variance, and thus, reliability [30]. Additionally, each task was considered clinically relevant, justifying that all tasks in the testing battery be retained [31].

### Intra-rater reliability

The high intra-rater agreement for observer 1 can be explained by the fact that the studied dogs had been her patients. On the other hand, high intra-rater reliability has also been found in expert observers evaluating motor function in human stroke patients [11] and forelimb locomotion in rats with experimental unilateral cervical spinal cord injury [32]. Observer 2 showed moderate agreement for ‘sitting’ (task 3), but almost perfect agreement for the other tasks. A re-check of the scoring sheets showed this observer to be consistently stricter in the second scoring for ‘sitting’ in most dogs. This could be explained by the video assessment and interpretation of the scoring criteria. In general, the observers found it challenging to, from the videos, distinguish between motivating the dog to maintain a desired position and providing support. The handler was stroking the dog on several occasions to calm it down. This could be interpreted as motivation to maintain position for the required time, but it could also be considered support because light support is defined as touching the dog < 5 times during the performance (Additional file 1). Evaluation of motor function from video recordings has the disadvantage, that the observer is able to evaluate only that exact performance, possibly missing details that would have been detectable in the live situation.

### Inter-rater reliability

Previous validated functional outcome measures have shown high inter-rater agreement [21, 22, 33]. The inter-rater reliability in this study was very promising, with little variance in the confidence intervals (Table 3) between observers, regardless of whether or not they were experienced. Relative to the FINFUN, the more brief TSCIS, a functional scale evaluating gait, proprioceptive positioning and nociception, showed substantial agreement in weighted kappa scores (0.72–01.00) and confidence intervals between moderate (0.42) and perfect (1.0). However, in their study the observers were of different educational levels and had received no training [21].

**Table 3:** Inter-rater reliability for individual tasks and the sum score of the testing battery

Task	Inter-rater experienced (n = 2) (95% CI)	Inter-rater novice (n = 5) (95% CI)	Inter-rater all (n = 7) (95% CI)
Lying	0.705 (0.341–0.868)	0.919 (0.857–0.959)	0.932 (0.881–0.966)
Standing up from lying	0.954 (0.896–0.980)	0.970 (0.947–0.985)	0.982 (0.969–0.991)
Sitting	0.758 (0.457–0.892)	0.697 (0.465–0.848)	0.793 (0.646–0.894)
Standing up from sitting	0.911 (0.638–0.968)	0.939 (0.885–0.970)	0.978 (0.962–0.989)
Standing	0.953 (0.881–0.980)	0.966 (0.940–0.983)	0.975 (0.958–0.987)
Proprioceptive positioning	0.988 (0.974–0.995)	0.968 (0.944–0.984)	0.983 (0.971–0.991)
Start to walk from standing	0.973 (0.941–0.988)	0.988 (0.978–0.994)	0.992 (0.985–0.996)
Walking	0.978 (0.951–0.990)	0.975 (0.956–0.987)	0.986 (0.976–0.993)
Running	0.977 (0.950–0.990)	0.962 (0.931–0.981)	0.978 (0.962–0.989)
Walking turns	0.968 (0.910–0.987)	0.967 (0.941–0.983)	0.981 (0.967–0.990)
Walking stairs	0.971 (0.936–0.987)	0.975 (0.956–0.988)	0.984 (0.972–0.992)
Sum score	0.993 (0.984–0.997)	0.993 (0.988–0.997)	0.996 (0.993–0.998)

The intra-class correlation coefficient and confidence intervals for the experienced and novice observers as well as for all observers combined. Data are shown for each individual task and for the FINFUN total score

Previous studies emphasize the importance of observer training when developing numerical scales to assess motor function since training reduces observer-related errors [11, 15, 34]. Novice observers may adapt quickly to scoring routines [17], and the observers volunteering for this study were involved also in the pilot study. Thus, they were familiar with the scoring process before undertaking the training, and this has certainly influenced the results positively. In accordance with previous reports, a learning curve was noted in this study [22]. The experiences from the pilot study and the training revealed that observers needed to practice the FINFUN at least eight times in order to feel comfortable in the scoring process. A similar amount of practice in the development of functional scoring in dogs with spinal cord injury has been reported [22]. Considering this, to achieve such high level of agreement as is presented here, the required practice scoring should be approximately 15 times.

Interestingly, the agreement is higher for the FINFUN sum score than for the separate tasks (Tables 2 and 3), indicating that observers agreed very well on the dogs’ overall function. However, there is variation > 5 points in the scores given for dogs 7, 10, 18, 24 and 25 (Table 1, dogs marked with an asterisk). These were dogs with good motor function (dogs 7, 10, 18 and 25) or very poor motor function (dog 24). Novice observers may not detect small details or mistakes in dogs’ motor function, therefore giving the dogs with good function a score of 4 (normal), whereas an experienced observer may be stricter, giving the same dog only a score of 3 (independent performance, mistakes occurring). This is in concordance with a previous study in which observers evaluating forelimb function in rats found that rating individuals with higher function was more difficult [32]. Increased observation time for specific periods and details may reduce the risk of missing important signs in the evaluation of locomotion [15]. Therefore, the FINFUN should be used so that the dog is allowed to perform the task more than once if needed and the best performance recorded. This gives the observer more time to decide on the score in the live situation, and this would correspond to the situation in clinical practice.

When validating a testing battery, it is of outmost importance that it is done under standardized conditions [12]. The frequently used video assessment ensures standardization in the evaluation of motor function [15, 23]. A recent study aiming to create a scoring system to detect the worse limb in dogs with thoracolumbar myelopathy found evaluations from video recordings to give higher inter-rater agreement than live evaluations [35]. In the current study, video assessment was chosen to reduce bias by enabling observers to assess the same performance of the dogs at the same time, excluding possible interfering factors from the environment, and thus, contributing to high reliability. This procedure also saved the patients the unnecessary stress of having several observers attending the therapy sessions. The dogs were filmed at the clinic in a standardized manner and the same person handled all the dogs during the video recordings. This was done to ensure that the handler would be as consistent as possible with amount of support or motivation for all the included dogs.

Although FINFUN showed high intra- and inter-rater reliability, it also has to be able to provide the practitioner with clinically relevant information. Based on the face and content validity we can argue that the FINFUN measures functional ability in the dog, providing scores that appear clinically relevant to the users in this study. The testing battery generated scores corresponding to the different grades of paralysis and no obvious floor or ceiling effect was noted. However, estimating the clinical relevance numerically with statistical tests was not within the scope of this study. This study focused on developing the testing battery itself and reliable scoring criteria. Still, the determination of the clinical relevance is a very important study that should follow the current one and further include determination of the sensitivity, specificity and responsiveness of the testing battery.

### Limitations

This study included only dogs with paraparesis or paraplegia, so this sample will not give variation in scoring of ‘lying’ (task 1), as perhaps would patients with tetraparesis. Most observers have scored ‘lying’ high (3 or 4), which may have influenced the overall results. Although different severities of paralysis were represented in the studied sample, no normal dogs were included. Therefore the sensitivity or specificity of the testing battery could not be evaluated. One of the observers (AB) was not blinded to the studied dogs, as they were her patients. By the time of the study, several months had passed since the video recordings. However, it cannot be excluded that not being blinded to the patients might have increased the reliability in the scoring for AB. The FINFUN does not distinguish between affected limbs, as does, for example, the TSCIS [21]. However the FINFUN allows possible discrepancy between limbs to be noted subjectively in the comments section. Considering the discussion above, the FINFUN scoring system may not be sensitive enough to evaluate the quality of near-normal movement. Therefore, the authors suggest the use of another validated scale focusing on assessment of walking quality [23, 24] simultaneously with the FINFUN, particularly when assessing already ambulatory patients.

The FINFUN is a tool designed to assess the overall function and quality of movement of canine patients to provide adequate information on the performance level of activities of daily living. It is to be used by veterinary physiotherapists working in the hospital setting, in both clinical practice and research. Further comparison with other, already validated, outcome measures should be carried out in future studies. Research regarding the construct validity of the FINFUN and its responsiveness to change in live dogs is underway.

## Conclusions

The FINFUN meets the demands of the growing field of physiotherapy and rehabilitation in veterinary medicine as an objective, valid and reliable tool with standardized scoring criteria for evaluation of motor function in dogs recovering from spinal cord injury.

## Additional file

**Additional file 1.** The Finnish neurological function testing battery for dogs.
