# Supplementary material for: Development of the Finnish neurological function testing battery for dogs and its intra- and inter-rater reliability
Source: Acta Vet Scand. 2018 Sep 17;60:56. doi: 10.1186/s13028-018-0408-2 (PMC6142623; doi:10.1186/s13028-018-0408-2)
Supplement: Supplementary file 1 — Additional file 1. The Finnish neurological function testing battery for dogs. [file 13028_2018_408_MOESM1_ESM.docx]

**Additional file 1**

**THE FINNISH NEUROLOGICAL FUNCTION TESTING BATTERY FOR DOGS (FINFUN)**

The Finnish Neurological Function Testing Battery for Dogs is a tool for evaluation of functional ability in dogs recovering from functional impairment caused by neurological disease. The testing battery is designed progressively such that tasks at the beginning of the test (1-6) require little functional ability to perform while tasks at the end (9-11) require greater functional ability to complete.

**Definitions of the terminology used in the testing battery**

**Support**

Support is defined as the method used to assist the dog during performance of a task. The support can be a towel, rope, sling or custom-made assistive device placed under the belly or between the affected limbs. Support can also be manual with the hands of the handler/assistant used to support the dog under the thorax, abdomen, laterally from the trunk or the hips or from the tail. The dog should be supported in a manner that allows the dog to perform the required task with the best possible functional ability and to achieve the highest possible score. The support must enhance the functional ability during the task and not restrict it. The handler should, however, avoid touching the dog unnecessarily during the performance.

*Strong support*: the dog needs support and assistance for the duration of the task; the handler supports most of the dog’s weight and/or touches the dog throughout the task.

*Light support:* the dog needs support occasionally during the task. The handler is allowed to touch/assist the dog a maximum of 5 times during the required task. If the handler touches/assists the dog more than 5 times during the task in question, this should be interpreted as strong support.

**Motivation**

The motivation is defined as the method used to encourage the dog to move in a desired direction at a desired moment, with the handler manually touching the dog as little as possible. The dog should be motivated in a manner that allows the dog to perform the required task with the best possible functional ability and to achieve the highest possible score. The motivation must enhance the functional ability during the task and not restrict it. Motivation can be a verbal command or a treat, a toy, another dog and/or a light pull from the lead/harness.

**Voluntary movements**

Voluntary movements are defined as the dog moving the affected limb(s) voluntarily, protraction of a limb with movement in at least one joint.

**Weight bearing of a limb/limbs**

Weight bearing is defined as the whole weight on the limb, all joints in functional weight bearing position during a minimum of two steps.

**Mistakes**

Mistakes include the dog falling, cross stepping, sidestepping, dragging paws and/or claws, or spontaneous over knuckling.

**Ataxia**

Ataxia is defined as lack of coordination without spasticity, paresis or involuntary movements, although each of these may be detected in relation to ataxia.

**Movement dysfunction**

Movement/locomotion dysfunction is defined as locomotion abnormal to the tested individual, e.g. lameness, bunny hopping, pacing (NOTE that pacing may be normal in some individuals and breeds).

**Compensatory movements**

Compensatory movements are defined as locomotor strategies used by the animal that are different to the ones used prior to injury/disease. There are various forms of compensation, ranging from mild modifications to complete replacement of a motor function.

**Weakness**

Weakness is defined as poor muscle strength, paralysis or general weakness due to abnormal general clinical status.

**Stress**

Stress is defined as the signs related to physical work, i.e. panting, restlessness, aroused behaviour, difficulties in maintaining the position and/or completing the task.

**GENERAL INSTRUCTIONS FOR THE ASSESSORS**

1. The assessor must become carefully acquainted with the testing criteria before using the testing battery.
2. The evaluation should be performed in the same area, in a standardized manner and with standardized equipment on every test occasion for the same dog.
3. The handler should be the same person for every task on every test occasion.
4. The dog should be rested before undertaking the test.
5. Tasks 1-6 are recommended to be performed inside and tasks 7-11 outside or on a non-slippery floor. The assessor must score the dog in the same place every time.
6. The support used is indicated in the “comments” section.
7. The motivation used (verbal commands, treats, toys, pull from lead/harness) is indicated in the “comments” section.
8. The dog should have an appropriate collar, lead or harness. The equipment is indicated in the “comments” section and should be the same on every test occasion.
9. The dog performs each task only once in order to avoid excessive stress on the dog. If the task must be repeated, the best performance is scored.
10. Restrictions related to surgery or disease that has been prescribed by the treating veterinary surgeon must be considered for each task during the test. They are indicated in the “comments” section.
11. Any medication (sedatives, NSAIDs, other analgesics, etc.) at the time of the test is recorded in the “comments” section.
12. Other diseases or problems (e.g. osteoarthritis) that may affect the performance are recorded in the “comments” section.
13. If the safety of the patient, handler, owner or assessor is threatened at any time during the test, the scoring is interrupted. The reason for the interruption is recorded in the “comments” section.
14. Every task is defined in the test criteria and the following scale is used for scoring each defined task:
15. Not able to perform the task at all or performs the task only partially and then strongly supported. No voluntary movements or only occasional movements in the affected limbs.
16. Completes the whole task or parts of the task strongly supported. Voluntary movements with no or only occasional weight bearing in affected limbs.
17. Completes the task with light support. The patient needs support occasionally. Voluntary movements and/or occasionally weight bearing in affected limbs.
18. Completes the task independently. Continuous weight bearing in affected limbs. The dog may show weakness/stress/poor muscle strength/ataxia and/or the dog makes mistakes.
19. Completes the task independently and effortlessly without weakness or mistakes. The performance is normal and/or at the same level as before paralysis.

**Guidelines to clarify the scoring**

An independent performance is more important during the scoring than support, motivation, number of mistakes or possible ataxia. The support needed is more important in the scoring than the amount of motivation, mistakes or possible ataxia. The number of mistakes is more important in the scoring than motivation or possible ataxia. If the assessor is unable to determine a score, the lower score should be given. The reason for this is indicated in the “comments” section.

**TEST CRITERIA**

1. **Lying sternal**

The dog is lying sternal and is able to maintain the position and follow the surrounding environment for 5 seconds. The hind limbs may be flexed closed to the body (lying square) or the limbs may be to one side.

- 1. **The dog is not able to lie in a sternal position at all.**
  2. **The dog is able to lie in a sternal position strongly supported.**

Occasional voluntary movements in one or several of the affected limbs.

- 1. **The dog is able to lie in a sternal position lightly supported.**

Occasional voluntary movements in one or several of the affected limbs.

- 1. **The dog is able to lie in a sternal position independently for 5 seconds.**

Weakness and/or stress visible, voluntary movements in one or several of the affected

limbs.

- 1. **The dog is able to lie in a sternal position for 5 seconds and follow the surrounding environment.**

No signs of weakness or stress. Voluntary movements in one or several of the affected

limbs.

1. **Standing up from lying**

The dog is lying on the floor/surface (sternal position or limbs to one side). The dog stands up from lying, using both hind limbs symmetrically according to visual evaluation, into a balanced standing position, and supporting weight symmetrically on the hind limbs. The dog is able to maintain the position for at least 3 seconds.

- 1. **The dog is not able to stand up from lying.**
  2. **The dog stands up from lying strongly supported.**
  3. **The dog stands up from lying lightly supported.**
  4. **The dog stands up from lying into standing position independently.**

The dog may show compensatory movements and/or asymmetrical use of hind limbs. The dog may pull itself up with its front limbs and/or may not able to maintain a balanced standing position for 3 seconds. The affected limbs may be left in an abnormal position, and the dog may show weakness and/or stress.

- 1. **The dog stands up from lying into a balanced standing position.**

The dog supports weight symmetrically on the hind limbs while standing up. The dog is able to maintain a balanced standing position for at least 3 seconds without apparent weakness or stress.

1. **Sitting**

The dog is sitting in a balanced position on the floor/surface for at least 5 seconds, with no support required. In visual evaluation, the dog supports weight symmetrically on both hind limbs; the dog is able to follow the environment. The position of the hind limbs is not important in criteria 0-3.

1. **The dog is not able to sit or maintain sitting position at all.**
2. **The dog is able to sit strongly supported.**
3. **The dog is able to sit lightly supported.**
4. **The dog is able to maintain sitting position independently for at least 5 seconds.**

Visible compensatory movements, weakness and/or stress.

1. **The dog is able to sit in a balanced position for at least 5 seconds.**

The weight is equally distributed on both hind limbs according to visual evaluation, and

the dog is able to follow the environment.

1. **Standing up from sitting**

The dog is sitting on the floor/surface in a balanced position, the weight symmetrically distributed on both hind limbs. The dog moves, using symmetrical thrust with both hind limbs, into a balanced standing position and is able to maintain this position for at least 3 seconds. The weight is visually symmetrically distributed on both the front and hind limbs.

1. **The dog is not able to stand up from sitting at all.**
2. **The dog stands up from sitting strongly supported.**
3. **The dog stands up from sitting and is able to maintain the standing position lightly supported.**
4. **The dog stands up from sitting independently and is able to independently maintain the standing position for at least 3 seconds.**

The dog may not use the hind limbs symmetrically when standing up and/or the dog may pull itself up using mainly the front limbs. The standing position may not be balanced (i.e. the weight may be distributed more to the front limbs or more to one limb). The dog may show weakness and/or stress.

1. **The dog stands up from sitting position into a balanced standing position, symmetrical thrust in both hind limbs.**

The dog is able to maintain the balanced standing position for at least 3 seconds.

**5. Standing**

The dog stands in a normal balanced standing position, the weight symmetrically distributed on front and hind limbs. The dog is able to maintain this position for at least 5 seconds.

1. **The dog is not able to stand at all.**
2. **The dog is able to stand strongly supported.**
3. **The dog is able to stand lightly supported for at least 5 seconds.**
4. **The dog is able to maintain in standing position for at least 5 seconds independently.**

The affected limbs may be in abnormal position and/or the weight unevenly distributed on the front and/or rear and/or on the front- or hind limbs, visible weakness and/or stress.

1. **The dog stands balanced for 5 seconds or more and is able to follow the environment.**

**6. Proprioceptive positioning**

Placing the dorsal surface of the dog’s foot against the ground tests the proprioceptive positioning. The dogs should replace the foot to its natural position immediately. The test is performed on all four limbs.

1. **The proprioceptive positioning is absent in all affected limbs.**
2. **The proprioceptive positioning is decreased in all affected limbs.**
3. **The proprioceptive positioning is mildly decreased in all affected limbs.**

(For the tetraparetic patient, the proprioceptive positioning is mildly decreased in three or four limbs)

1. **The proprioceptive positioning is normal in one of the affected limbs.**

(For a paraparetic patient, the proprioceptive positioning is decreased in only one limb. For the tetraparetic patient, the proprioceptive positioning is decreased in only one or two limbs)

1. **The proprioceptive positioning is normal in all four limbs.**

**7. Start walking from a balanced standing position.**

The dog stands in a normal balanced standing position. The dog is asked to walk forward three metres.

1. **The dog needs heavy support in standing, is not able to start walking without strong support, may fall.**

Non-ambulatory paraparesis. No or occasional voluntary movements in the affected limbs, no weight bearing in the affected limbs.

1. **The dog stands with light support, needs strong support when walking.**

Non-ambulatory paraparesis. Occasional or continuous voluntary movements in the affected limbs, no or occasional weight bearing in the affected limbs.

1. **The dog stands independently, needs light support when walking.**

Non-ambulatory to ambulatory paraparesis. Continuous voluntary movements in the affected limbs, occasional weight bearing in affected limbs, not strong enough to walk 3 metres.

1. **The dog stands and starts to walk independently, walks 3 metres.**

Ambulatory paraparesis. Continuous weight bearing in affected limbs. The dog may show weakness and/or stress, and/or ataxia, and/or mistakes.

1. **The dog starts to walk from a normal/near-normal standing position, and walks 3 metres or more.**

No visible weakness, stress, ataxia or mistakes.

**8. Walking**

The dog walks at least 30 metres back and forth (altogether 60 metres).

1. **The dog is not able to walk 30 metres.**

Non-ambulatory paraparesis. Occasional voluntary movements in the affected limbs.

1. **The dog walks 30 metres or less, strongly supported.**

Non-ambulatory paraparesis. Voluntary movements in the affected limbs, occasional weight bearing in the affected limbs, visible weakness and/or stress.

1. **The dog walks more than 30 metres, lightly supported.**

Ambulatory paraparesis with occasional or continuous weight bearing in the affected limbs. The dog may show weakness and/or stress, and/or compensatory movements and/or ataxia. More than 5 mistakes occur.

1. **The dog walks 30 metres or more, independently.**

Ambulatory paraparesis with continuous weight bearing in the affected limbs. The dog may show weakness and/or stress and/or compensatory movements and/or ataxia. Less than 5 mistakes occur.

1. **The dog walks 30 metres back and forth (altogether 60 metres).**

The walking is normal/near-normal, muscle strength is normal and walking movement is at the level prior to paralysis.

**9. Running**

The dog is asked to run at least 30 metres back and forth (altogether 60 metres).

1. **The dog is not able to run at all.**

May take a few steps, but falls immediately.

1. **The dog is able to run with light support 30 metres or less.**

The dog may show weakness and/or stress and/or compensatory movements due to ataxia. More than 5 mistakes occur.

1. **The dog runs 30 metres or more, but struggles to do it independently.**

The dog may show compensatory movements and/or ataxia. Less than 5 mistakes occur.

1. **The dog runs 30 metres independently.**

The dogs may show ataxia and/or compensatory movements, but no mistakes occur.

1. **The dog runs 30 metres back and forth effortlessly (altogether 60 metres).**

The dog runs at trotting speed or faster, movement is normal/near-normal; muscle strength is normal and/or movement is at the same level as before paralysis.

**10. Walking turns**

The dog is walked briskly on a tight figure eight around the handler. The handler is instructed to walk the dog on the smallest figure eight possible.

1. **The dog is able to walk with light support on a straight line.**

Falls in the turns and needs assistance to get back up*.*

1. **The dog is able to walk the turns with light support, may fall in both directions.**

Gets up independently. The dogs may show weakness and/or stress and/or compensatory movement and/or ataxia. More than 5 mistakes occur.

1. **The dog walks the turns independently, may fall in one direction.**

Gets up independently. The dogs may show weakness and/or stress and/or compensatory movement and/or ataxia. More than 5 mistakes occur.

1. **The dog walks the turns in both directions independently without falling.**

The dogs may show compensatory movements and/or ataxia. Less than 5 mistakes occur.

1. **The dog walks the turns independently in both directions effortlessly.**

No weakness, stress, mistakes or ataxia. Movement is at the level as before paralysis.

**11. Stair climbing**

The dog is asked to climb up and down the stairs. The dog is allowed to rest for a maximum of 30 seconds at the top of the stairs.

**0. The dog is not able to climb the stairs at all.**

Non-ambulatory paraparesis. Occasional voluntary movements, but no or occasional weight bearing in the affected limbs, visible weakness and/or stress.

1. **The dog climbs the stairs strongly supported.**

Non-ambulatory paraparesis to ambulatory paraparesis. Occasional or continuous weight bearing in affected limbs. The dogs may show weakness and/or stress.

1. **The dog climbs up and down the stairs, needing support climbing up or down.**

Ambulatory paraparesis. The dog may show weakness and/or stress. More than 5 mistakes occur.

1. **The dog climbs up and down the stairs independently.**

The dog may show weakness and/or stress and/or compensatory movements and/or ataxia. Less than 5 mistakes occur.

1. **The dog climbs up and down the stairs effortlessly.**

The movement is normal/near-normal or at the level before paralysis

**THE FINNISH NEUROLOGICAL FUNCTION TESTING BATTERY FOR DOGS**

**SCORING SHEET**

| **Owner** |  | **Date/time** |  |
| --- | --- | --- | --- |
| **Animal** |  | **Breed** |  |
| **Observer** |  | **Diagnosis** |  |

| **Task** | **Score** | **Comments** |
| --- | --- | --- |
| 1. **Lying sternal** |  |  |
| 1. **Standing up from lying** |  |  |
| 1. **Sitting** |  |  |
| 1. **Standing up from sitting** |  |  |
| 1. **Standing** |  |  |
| 1. **Proprioceptive positioning** |  |  |
| 1. **Start to walk from standing** |  |  |
| 1. **Walking** |  |  |
| 1. **Running** |  |  |
| 1. **Walking turns** |  |  |
| 1. **Walking stairs** |  |  |
| **Sum score** |  |  |
